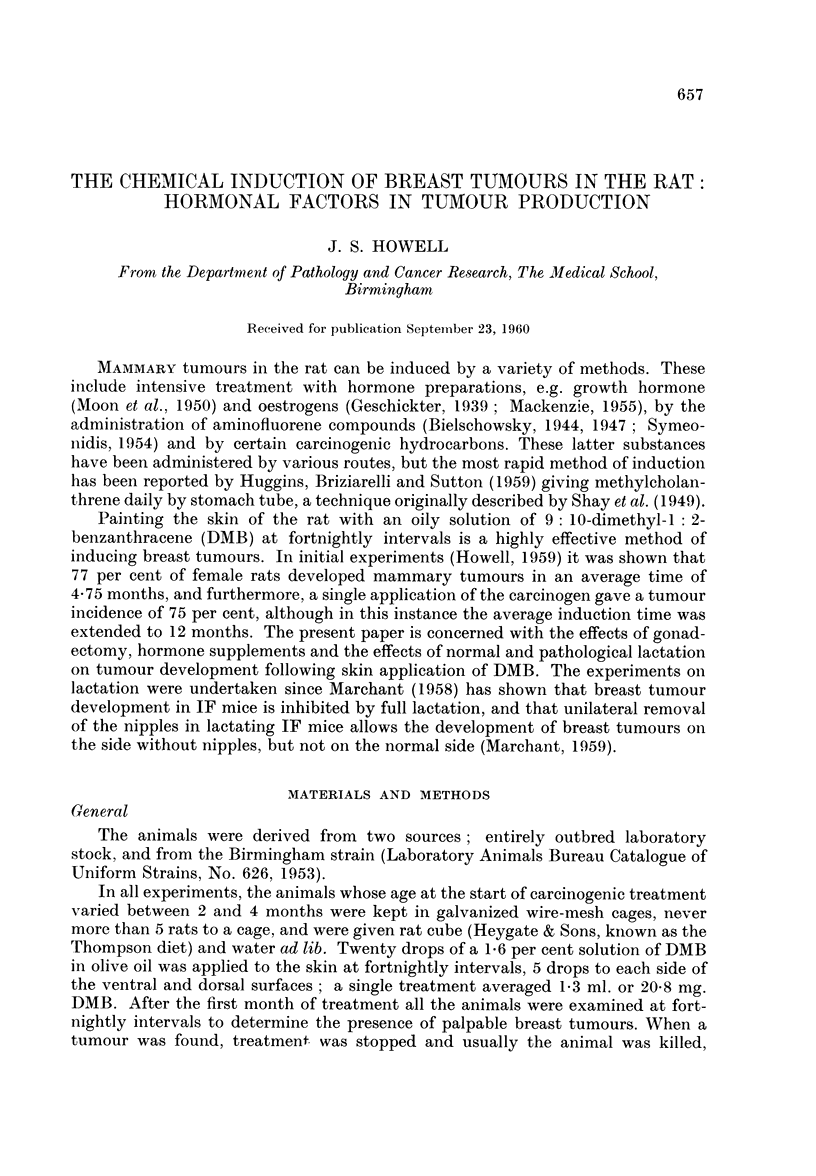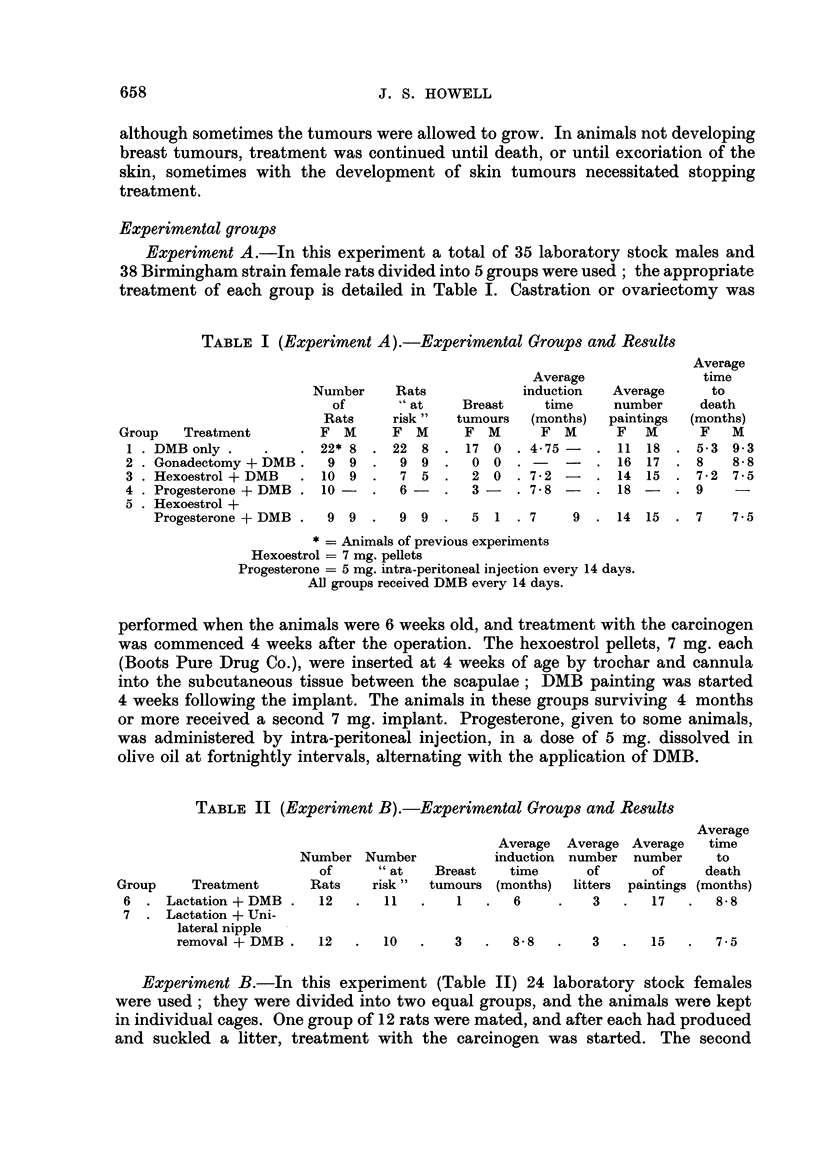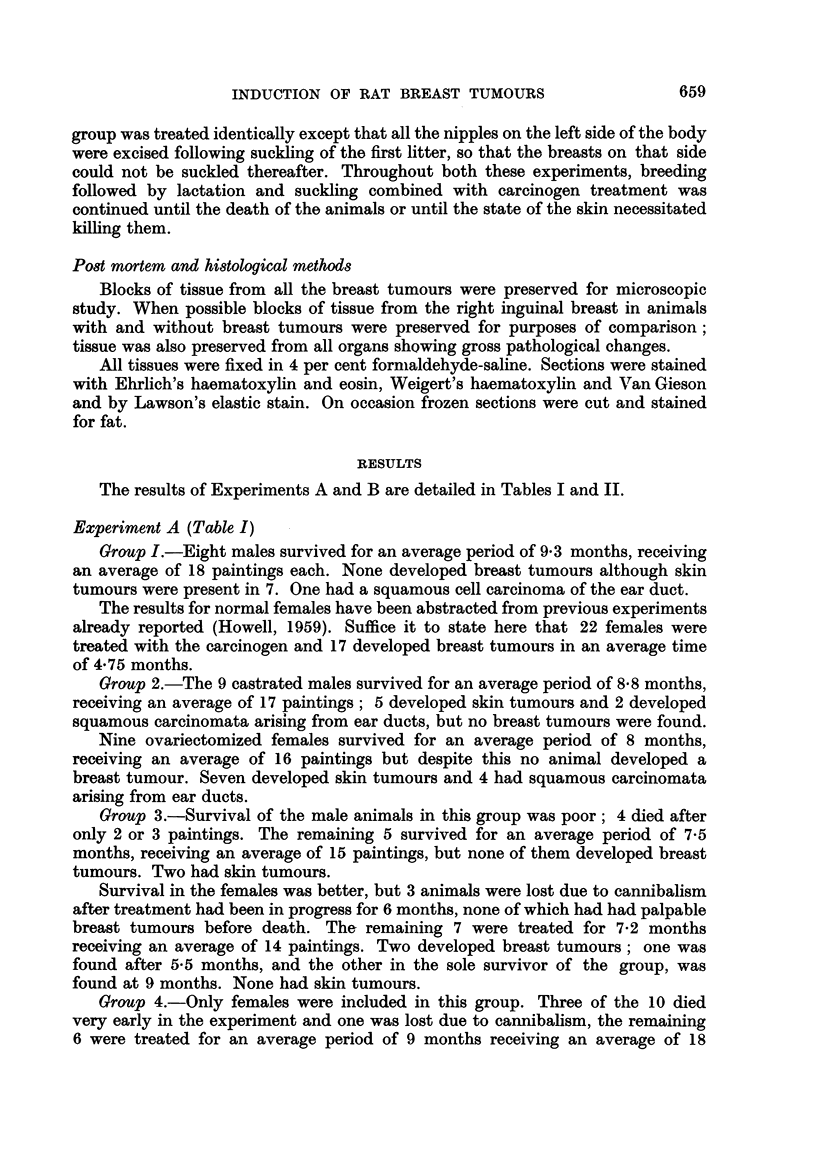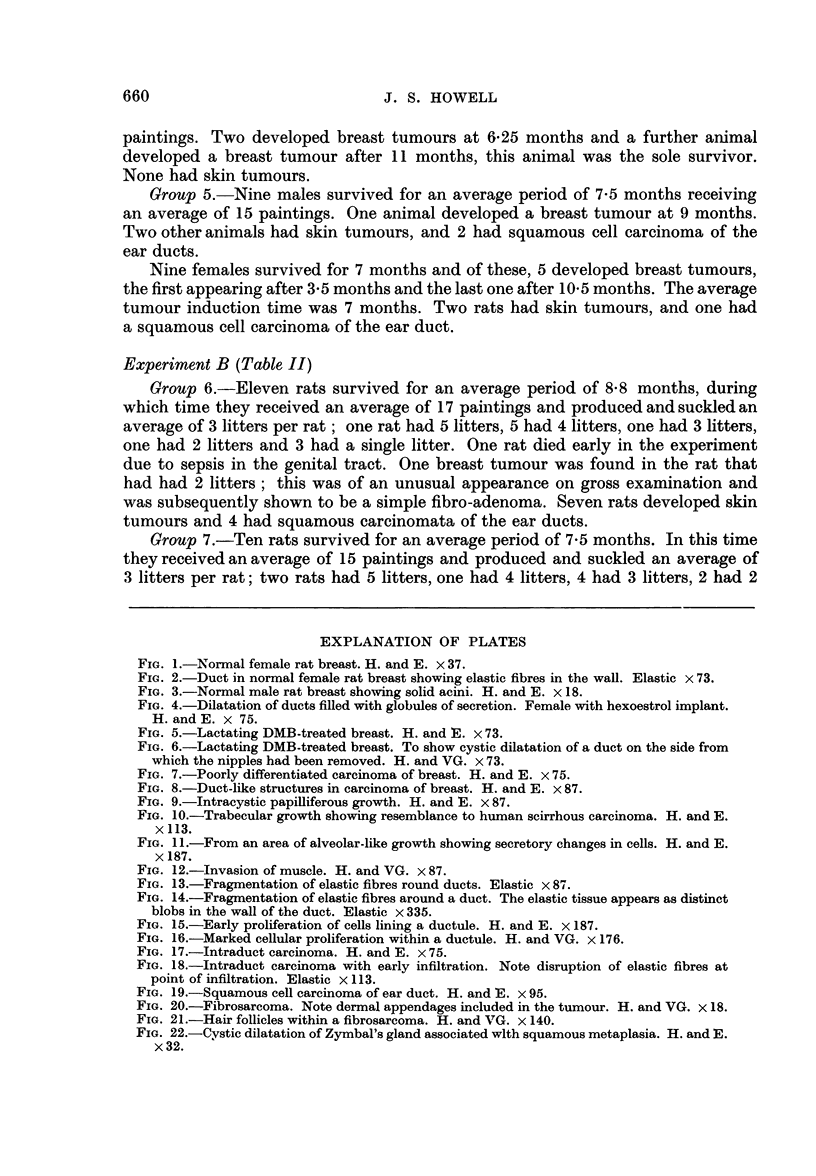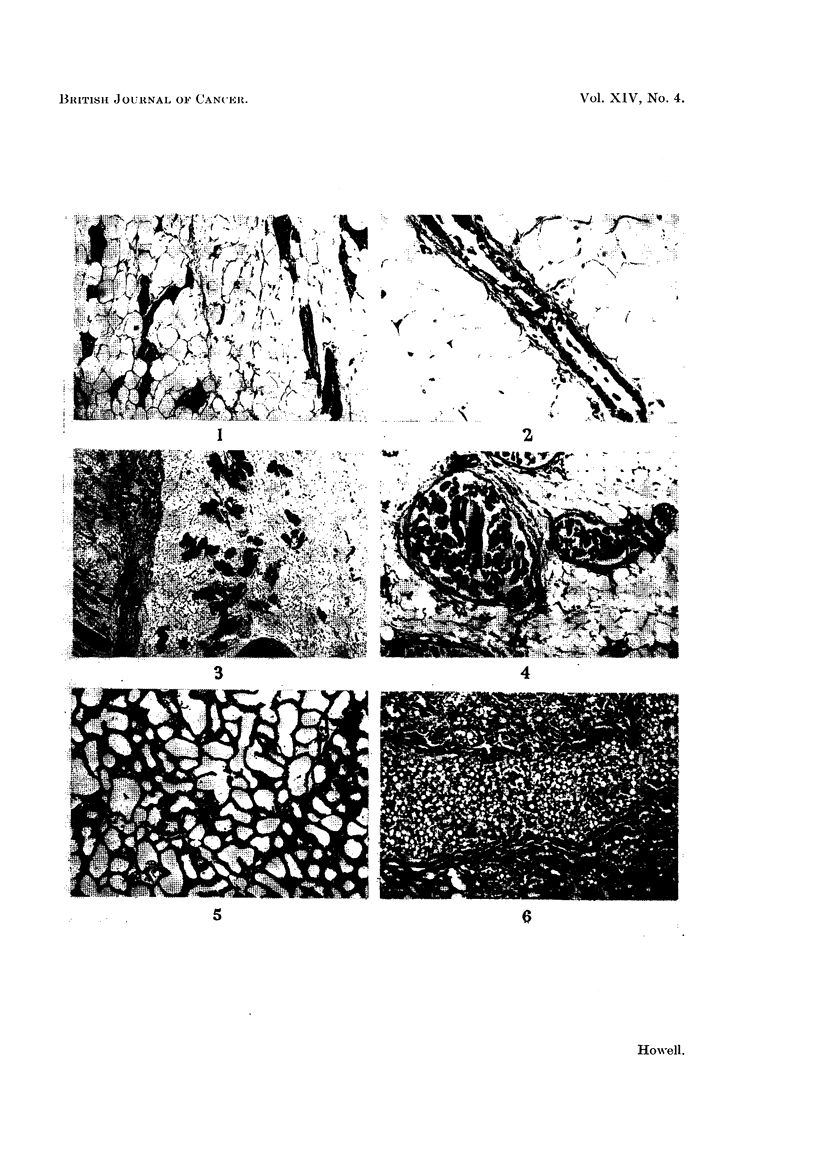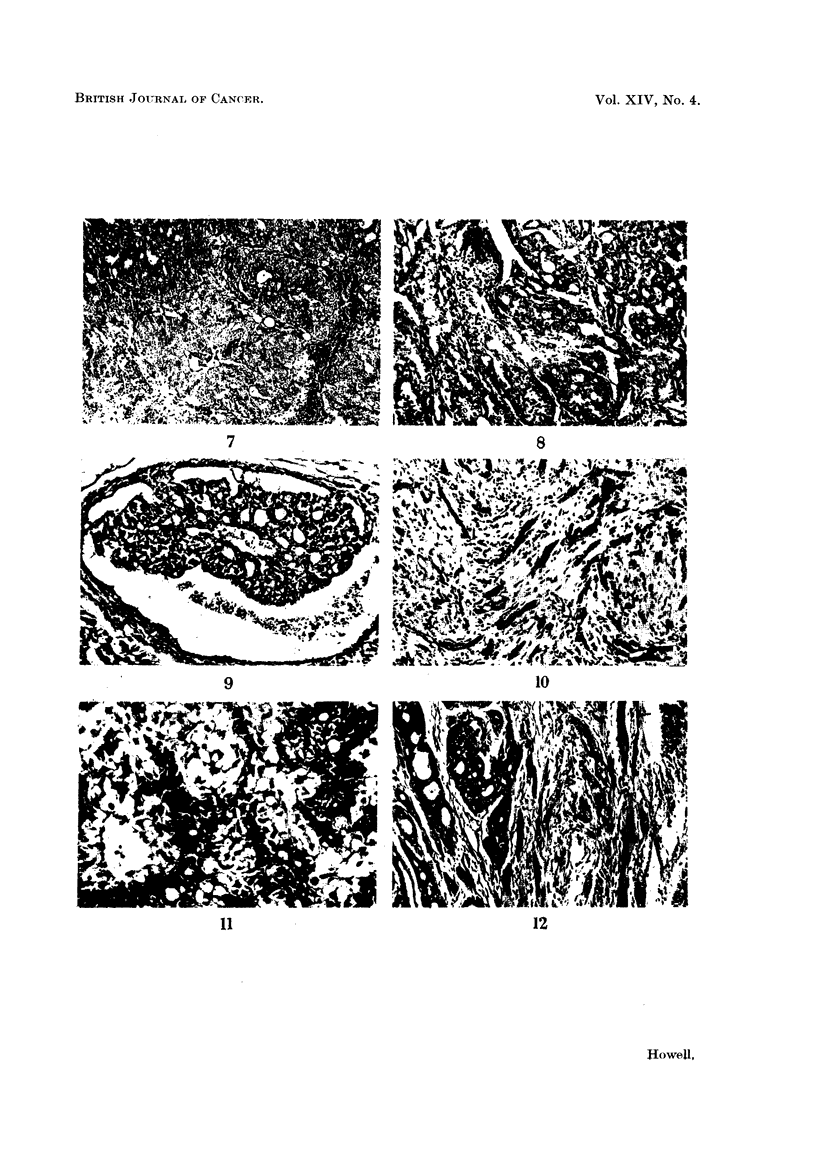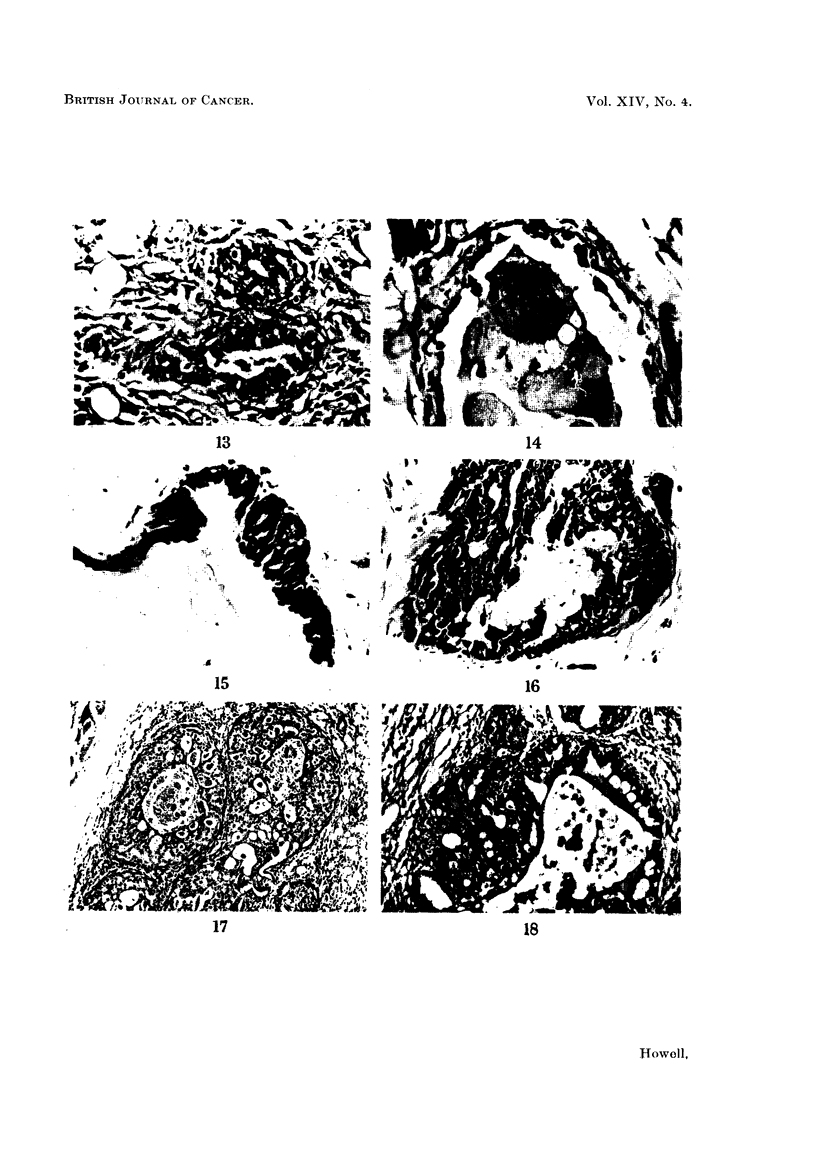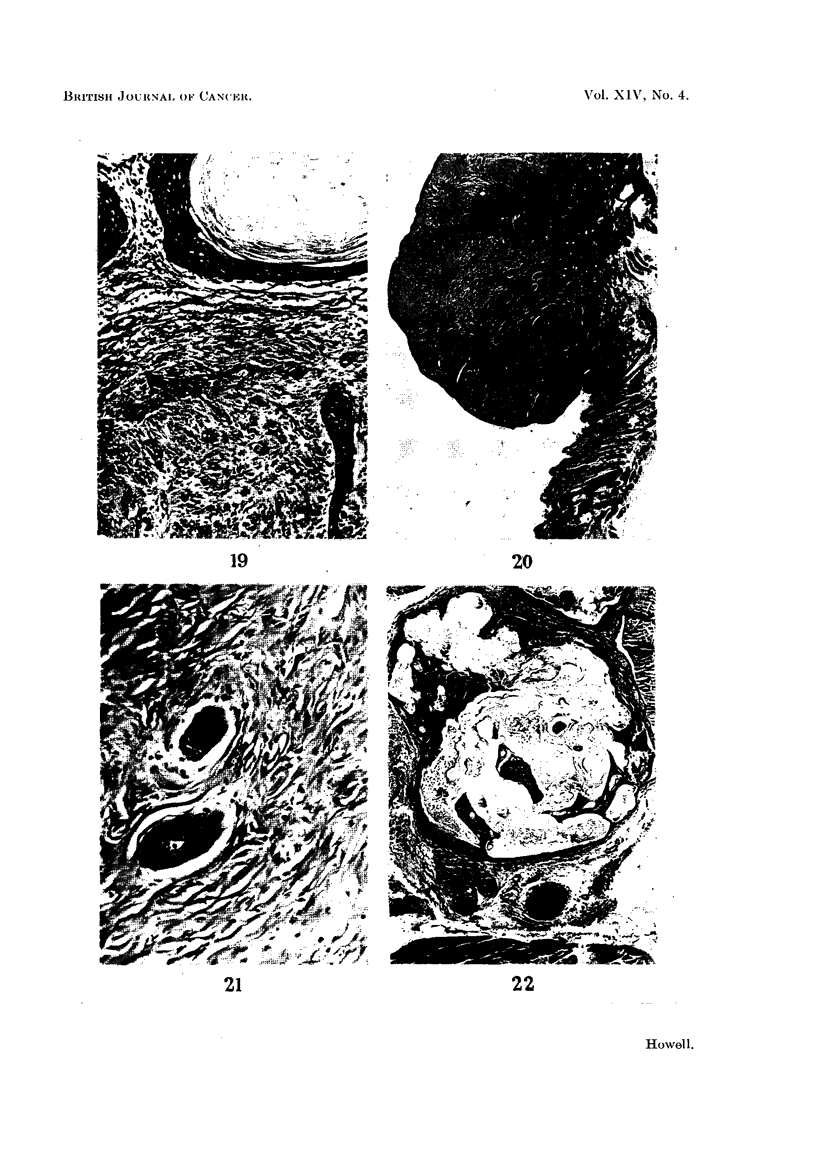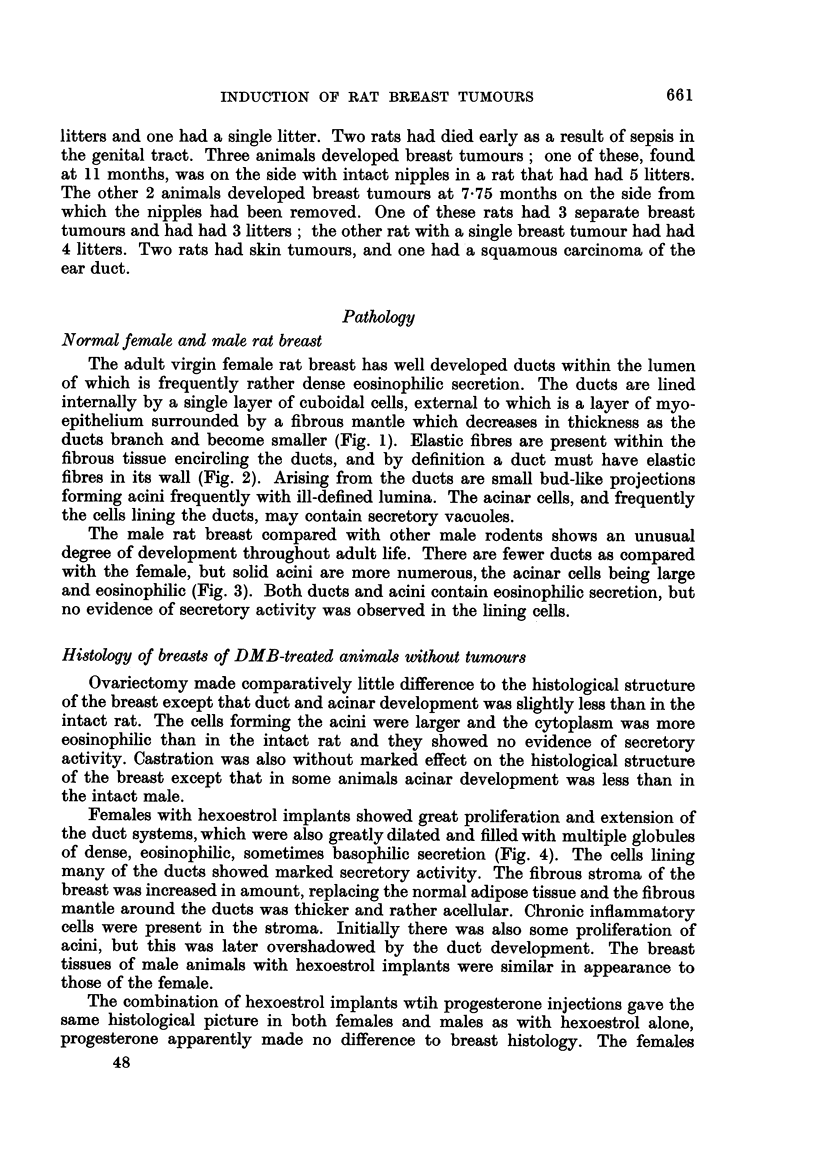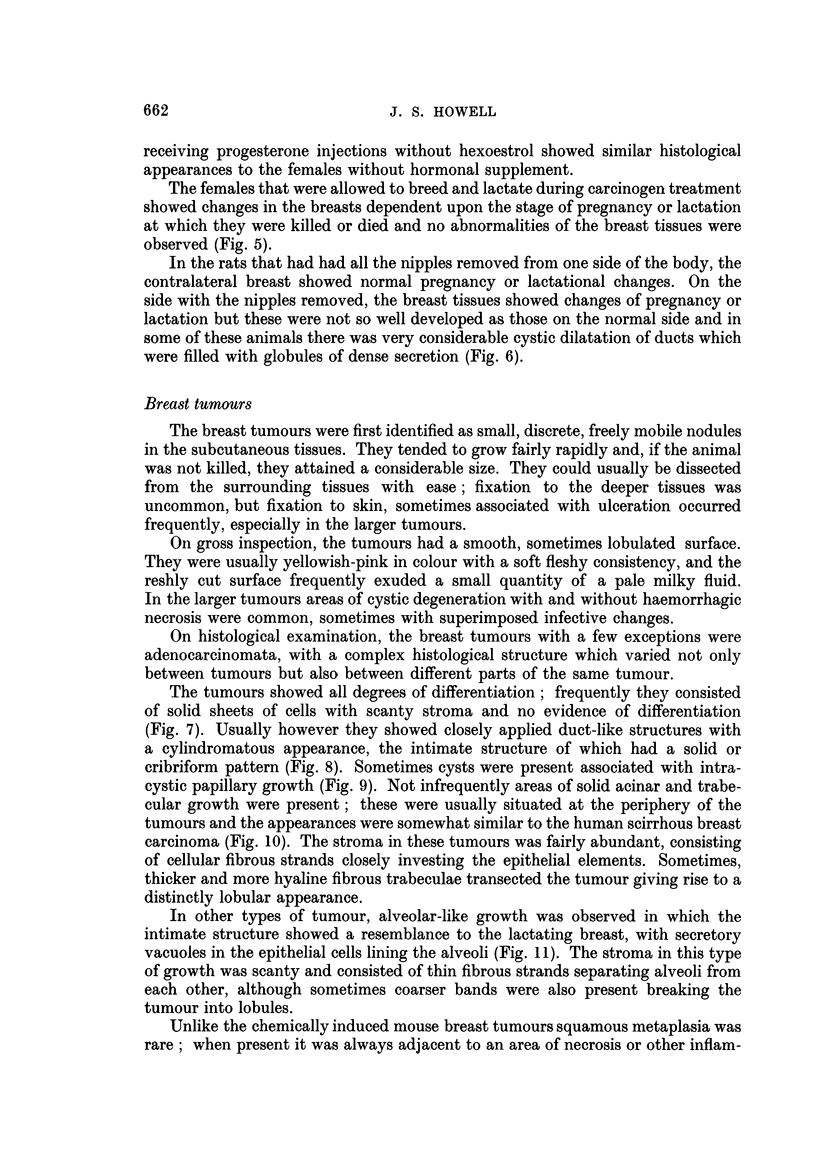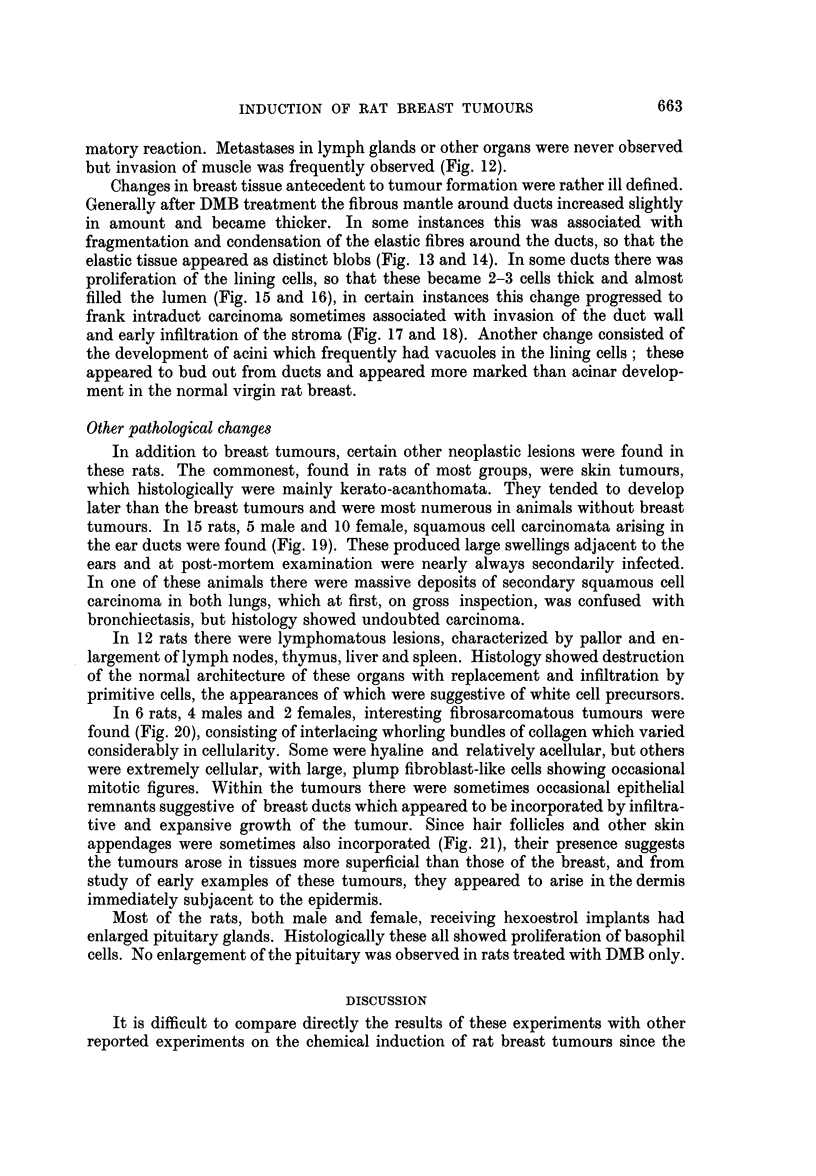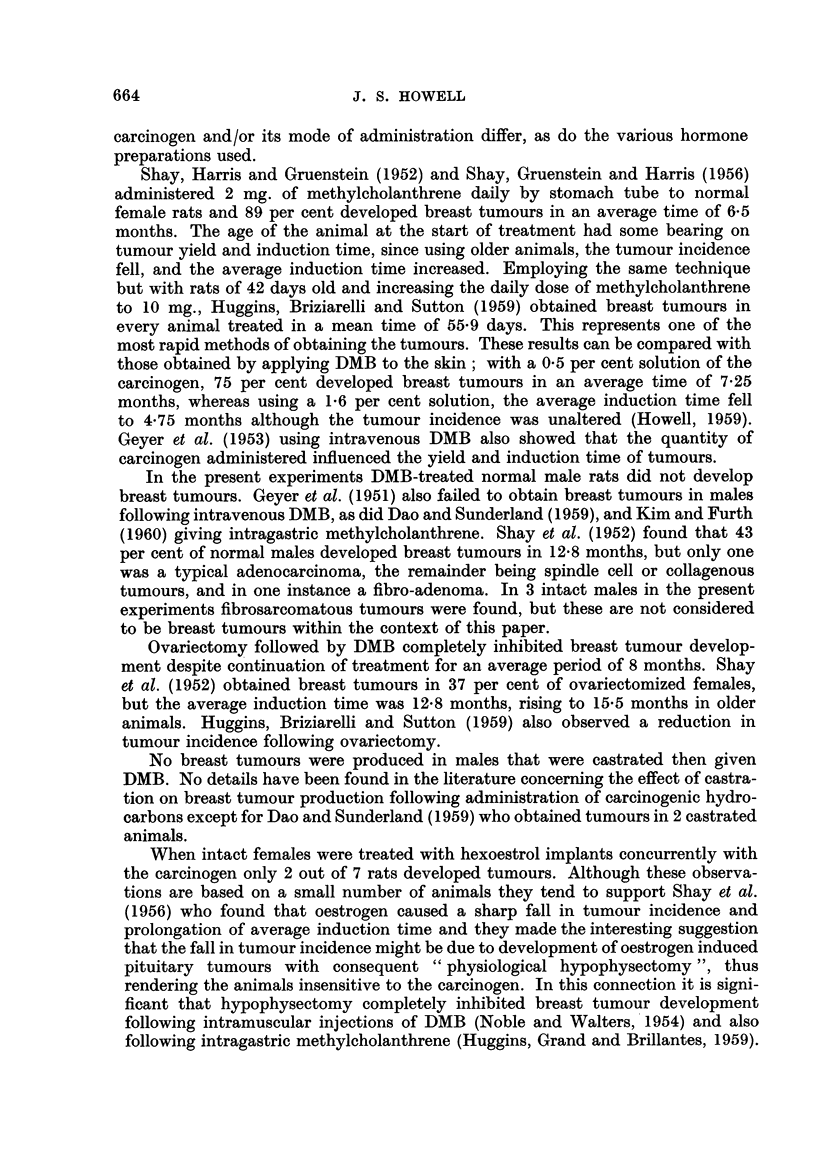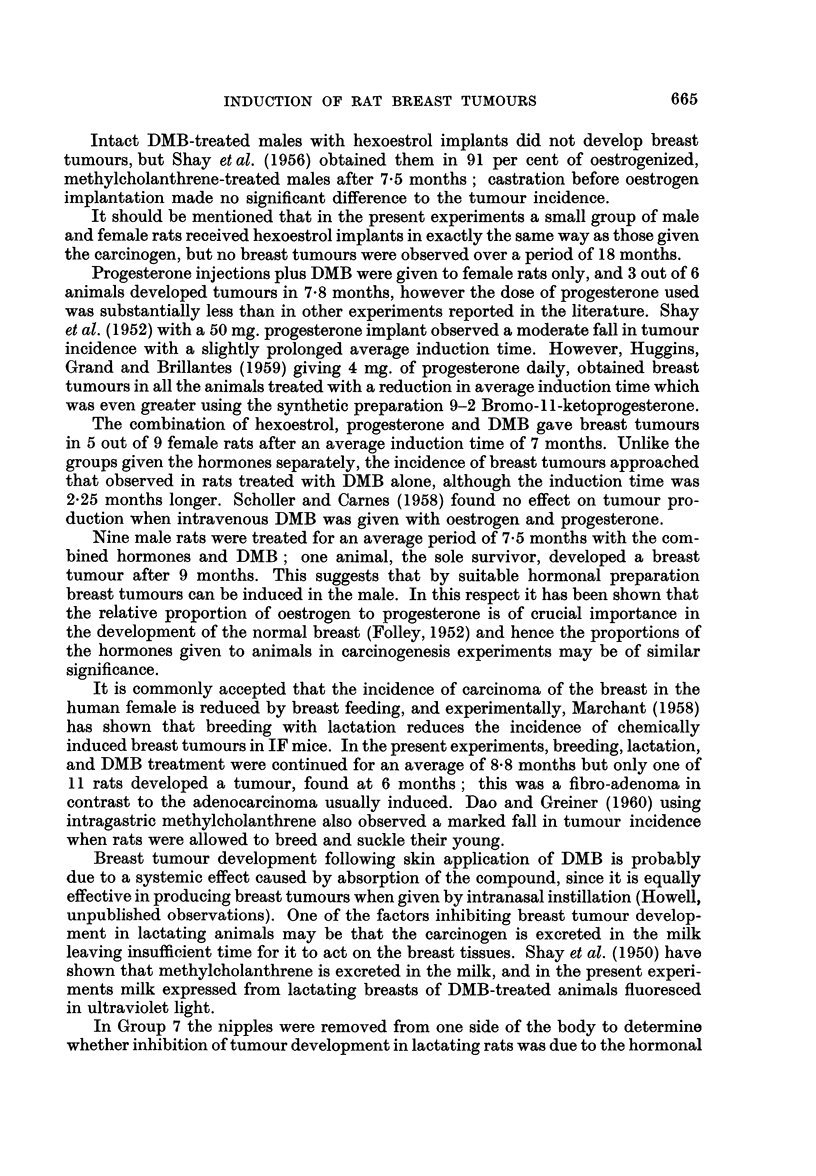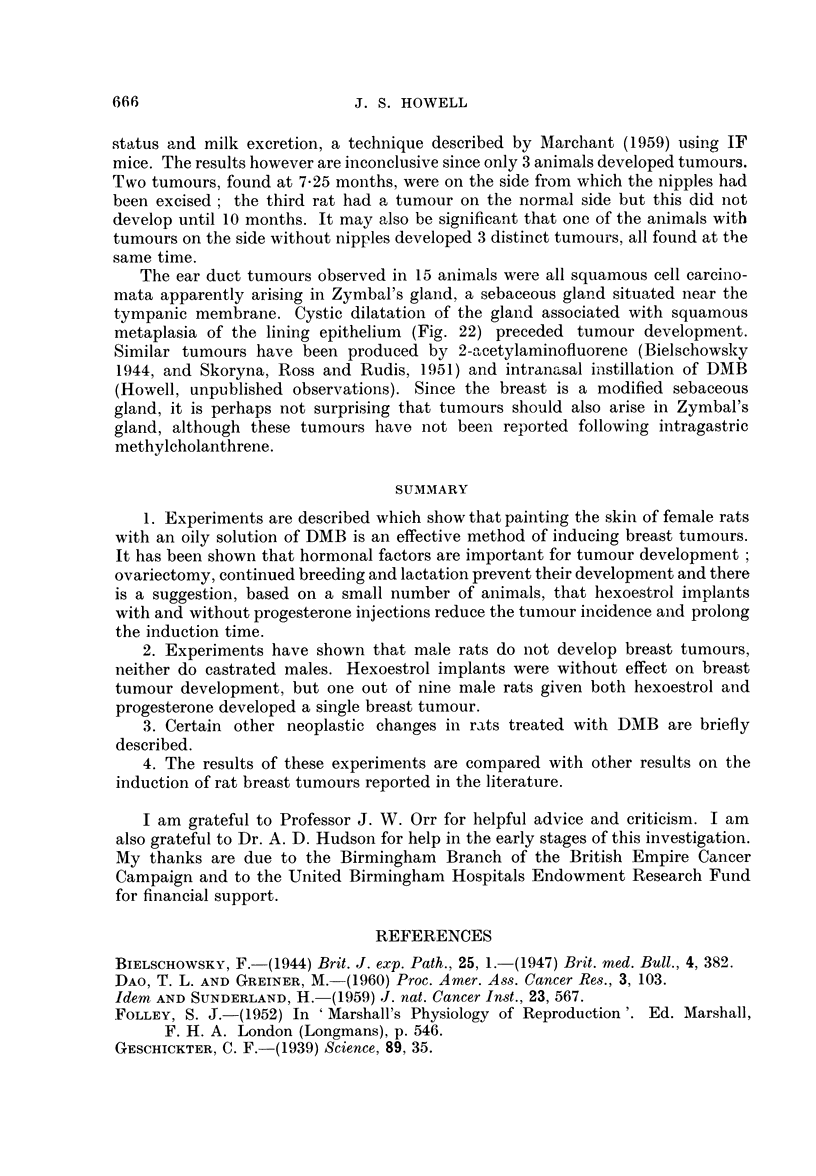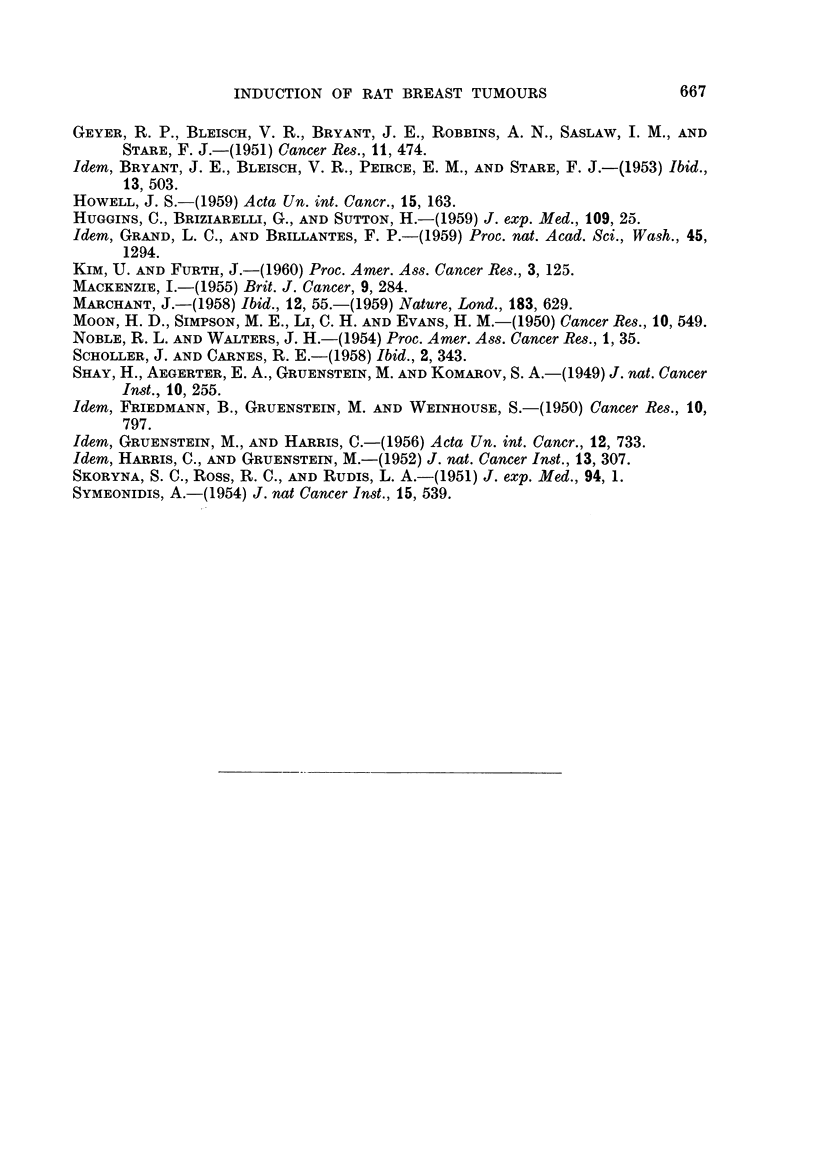# The Chemical Induction of Breast Tumours in the Rat: Hormonal Factors in Tumour Production

**DOI:** 10.1038/bjc.1960.73

**Published:** 1960-12

**Authors:** J. S. Howell

## Abstract

**Images:**


### THE CHEMICAL INDUCTION OF BREAST TUMOURS IN THE RAT: HORMONAL FACTORS IN TUMOUR PRODUCTION

J. S. HOWELL

*From the Department of Pathology and Cancer Research, The Medical School, Birmingham*

Received for publication September 23, 1960

MAMMARY tumours in the rat can be induced by a variety of methods. These include intensive treatment with hormone preparations, e.g. growth hormone (Moon *et al.*, 1950) and oestrogens (Geschickter, 1939; Mackenzie, 1955), by the administration of aminofluorene compounds (Bielschowsky, 1944, 1947; Symeonidis, 1954) and by certain carcinogenic hydrocarbons. These latter substances have been administered by various routes, but the most rapid method of induction has been reported by Huggins, Briziarelli and Sutton (1959) giving methylcholanthrene daily by stomach tube, a technique originally described by Shay *et al.* (1949).

Painting the skin of the rat with an oily solution of 9 : 10-dimethyl-1 : 2-benzanthracene (DMB) at fortnightly intervals is a highly effective method of inducing breast tumours. In initial experiments (Howell, 1959) it was shown that 77 per cent of female rats developed mammary tumours in an average time of 4·75 months, and furthermore, a single application of the carcinogen gave a tumour incidence of 75 per cent, although in this instance the average induction time was extended to 12 months. The present paper is concerned with the effects of gonadectomy, hormone supplements and the effects of normal and pathological lactation on tumour development following skin application of DMB. The experiments on lactation were undertaken since Marchant (1958) has shown that breast tumour development in IF mice is inhibited by full lactation, and that unilateral removal of the nipples in lactating IF mice allows the development of breast tumours on the side without nipples, but not on the normal side (Marchant, 1959).

### MATERIALS AND METHODS

### *General*

The animals were derived from two sources; entirely outbred laboratory stock, and from the Birmingham strain (Laboratory Animals Bureau Catalogue of Uniform Strains, No. 626, 1953).

In all experiments, the animals whose age at the start of carcinogenic treatment varied between 2 and 4 months were kept in galvanized wire-mesh cages, never more than 5 rats to a cage, and were given rat cube (Heygate & Sons, known as the Thompson diet) and water *ad lib.* Twenty drops of a 1·6 per cent solution of DMB in olive oil was applied to the skin at fortnightly intervals, 5 drops to each side of the ventral and dorsal surfaces; a single treatment averaged 1·3 ml. or 20·8 mg. DMB. After the first month of treatment all the animals were examined at fortnightly intervals to determine the presence of palpable breast tumours. When a tumour was found, treatment was stopped and usually the animal was killed, although sometimes the tumours were allowed to grow. In animals not developing breast tumours, treatment was continued until death, or until excoriation of the skin, sometimes with the development of skin tumours necessitated stopping treatment.

*Experimental groups*

*Experiment A*.—In this experiment a total of 35 laboratory stock males and 38 Birmingham strain female rats divided into 5 groups were used ; the appropriate treatment of each group is detailed in Table I. Castration or ovariectomy was performed when the animals were 6 weeks old, and treatment with the carcinogen was commenced 4 weeks after the operation. The hexoestrol pellets, 7 mg. each (Boots Pure Drug Co.), were inserted at 4 weeks of age by trochar and cannula into the subcutaneous tissue between the scapulae ; DMB painting was started 4 weeks following the implant. The animals in these groups surviving 4 months or more received a second 7 mg. implant. Progesterone, given to some animals, was administered by intra-peritoneal injection, in a dose of 5 mg. dissolved in olive oil at fortnightly intervals, alternating with the application of DMB.

TABLE I (*Experiment A*).—*Experimental Groups and Results*

| Group | Treatment | Number of Rats F | Number of Rats M | Rats "at risk" F | Rats "at risk" M | Breast tumours F | Breast tumours M | Average induction time (months) F | Average induction time (months) M | Average number paintings F | Average number paintings M | Average time to death (months) F | Average time to death (months) M |
|---|---|---|---|---|---|---|---|---|---|---|---|---|---|
| 1 | DMB only | 22* | 8 | 22 | 8 | 17 | 0 | 4·75 | — | 11 | 18 | 5·3 | 9·3 |
| 2 | Gonadectomy + DMB | 9 | 9 | 9 | 9 | 0 | 0 | — | — | 16 | 17 | 8 | 8·8 |
| 3 | Hexoestrol + DMB | 10 | 9 | 7 | 5 | 2 | 0 | 7·2 | — | 14 | 15 | 7·2 | 7·5 |
| 4 | Progesterone + DMB | 10 | — | 6 | — | 3 | — | 7·8 | — | 18 | — | 9 | — |
| 5 | Hexoestrol + Progesterone + DMB | 9 | 9 | 9 | 9 | 5 | 1 | 7 | 9 | 14 | 15 | 7 | 7·5 |

* = Animals of previous experiments
Hexoestrol = 7 mg. pellets
Progesterone = 5 mg. intra-peritoneal injection every 14 days.
All groups received DMB every 14 days.

TABLE II (*Experiment B*).—*Experimental Groups and Results*

| Group | Treatment | Number of Rats | Number "at risk" | Breast tumours | Average induction time (months) | Average number of litters | Average number of paintings | Average time to death (months) |
|---|---|---|---|---|---|---|---|---|
| 6 | Lactation + DMB | 12 | 11 | 1 | 6 | 3 | 17 | 8·8 |
| 7 | Lactation + Unilateral nipple removal + DMB | 12 | 10 | 3 | 8·8 | 3 | 15 | 7·5 |

*Experiment B*.—In this experiment (Table II) 24 laboratory stock females were used ; they were divided into two equal groups, and the animals were kept in individual cages. One group of 12 rats were mated, and after each had produced and suckled a litter, treatment with the carcinogen was started. The second group was treated identically except that all the nipples on the left side of the body were excised following suckling of the first litter, so that the breasts on that side could not be suckled thereafter. Throughout both these experiments, breeding followed by lactation and suckling combined with carcinogen treatment was continued until the death of the animals or until the state of the skin necessitated killing them.

*Post mortem and histological methods*

Blocks of tissue from all the breast tumours were preserved for microscopic study. When possible blocks of tissue from the right inguinal breast in animals with and without breast tumours were preserved for purposes of comparison; tissue was also preserved from all organs showing gross pathological changes.

All tissues were fixed in 4 per cent formaldehyde-saline. Sections were stained with Ehrlich's haematoxylin and eosin, Weigert's haematoxylin and Van Gieson and by Lawson's elastic stain. On occasion frozen sections were cut and stained for fat.

### RESULTS

The results of Experiments A and B are detailed in Tables I and II.

*Experiment A* (*Table I*)

*Group I*.—Eight males survived for an average period of 9·3 months, receiving an average of 18 paintings each. None developed breast tumours although skin tumours were present in 7. One had a squamous cell carcinoma of the ear duct.

The results for normal females have been abstracted from previous experiments already reported (Howell, 1959). Suffice it to state here that 22 females were treated with the carcinogen and 17 developed breast tumours in an average time of 4·75 months.

*Group* 2.—The 9 castrated males survived for an average period of 8·8 months, receiving an average of 17 paintings; 5 developed skin tumours and 2 developed squamous carcinomata arising from ear ducts, but no breast tumours were found.

Nine ovariectomized females survived for an average period of 8 months, receiving an average of 16 paintings but despite this no animal developed a breast tumour. Seven developed skin tumours and 4 had squamous carcinomata arising from ear ducts.

*Group* 3.—Survival of the male animals in this group was poor; 4 died after only 2 or 3 paintings. The remaining 5 survived for an average period of 7·5 months, receiving an average of 15 paintings, but none of them developed breast tumours. Two had skin tumours.

Survival in the females was better, but 3 animals were lost due to cannibalism after treatment had been in progress for 6 months, none of which had had palpable breast tumours before death. The remaining 7 were treated for 7·2 months receiving an average of 14 paintings. Two developed breast tumours; one was found after 5·5 months, and the other in the sole survivor of the group, was found at 9 months. None had skin tumours.

*Group* 4.—Only females were included in this group. Three of the 10 died very early in the experiment and one was lost due to cannibalism, the remaining 6 were treated for an average period of 9 months receiving an average of 18

paintings. Two developed breast tumours at 6·25 months and a further animal developed a breast tumour after 11 months, this animal was the sole survivor. None had skin tumours.

*Group* 5.—Nine males survived for an average period of 7·5 months receiving an average of 15 paintings. One animal developed a breast tumour at 9 months. Two other animals had skin tumours, and 2 had squamous cell carcinoma of the ear ducts.

Nine females survived for 7 months and of these, 5 developed breast tumours, the first appearing after 3·5 months and the last one after 10·5 months. The average tumour induction time was 7 months. Two rats had skin tumours, and one had a squamous cell carcinoma of the ear duct.

*Experiment B* (*Table II*)

*Group* 6.—Eleven rats survived for an average period of 8·8 months, during which time they received an average of 17 paintings and produced and suckled an average of 3 litters per rat ; one rat had 5 litters, 5 had 4 litters, one had 3 litters, one had 2 litters and 3 had a single litter. One rat died early in the experiment due to sepsis in the genital tract. One breast tumour was found in the rat that had had 2 litters ; this was of an unusual appearance on gross examination and was subsequently shown to be a simple fibro-adenoma. Seven rats developed skin tumours and 4 had squamous carcinomata of the ear ducts.

*Group* 7.—Ten rats survived for an average period of 7·5 months. In this time they received an average of 15 paintings and produced and suckled an average of 3 litters per rat; two rats had 5 litters, one had 4 litters, 4 had 3 litters, 2 had 2

---

EXPLANATION OF PLATES

FIG. 1.—Normal female rat breast. H. and E. ×37.
FIG. 2.—Duct in normal female rat breast showing elastic fibres in the wall. Elastic ×73.
FIG. 3.—Normal male rat breast showing solid acini. H. and E. ×18.
FIG. 4.—Dilatation of ducts filled with globules of secretion. Female with hexoestrol implant. H. and E. × 75.
FIG. 5.—Lactating DMB-treated breast. H. and E. ×73.
FIG. 6.—Lactating DMB-treated breast. To show cystic dilatation of a duct on the side from which the nipples had been removed. H. and VG. ×73.
FIG. 7.—Poorly differentiated carcinoma of breast. H. and E. ×75.
FIG. 8.—Duct-like structures in carcinoma of breast. H. and E. ×87.
FIG. 9.—Intracystic papilliferous growth. H. and E. ×87.
FIG. 10.—Trabecular growth showing resemblance to human scirrhous carcinoma. H. and E. ×113.
FIG. 11.—From an area of alveolar-like growth showing secretory changes in cells. H. and E. ×187.
FIG. 12.—Invasion of muscle. H. and VG. ×87.
FIG. 13.—Fragmentation of elastic fibres round ducts. Elastic ×87.
FIG. 14.—Fragmentation of elastic fibres around a duct. The elastic tissue appears as distinct blobs in the wall of the duct. Elastic ×335.
FIG. 15.—Early proliferation of cells lining a ductule. H. and E. ×187.
FIG. 16.—Marked cellular proliferation within a ductule. H. and VG. ×176.
FIG. 17.—Intraduct carcinoma. H. and E. ×75.
FIG. 18.—Intraduct carcinoma with early infiltration. Note disruption of elastic fibres at point of infiltration. Elastic ×113.
FIG. 19.—Squamous cell carcinoma of ear duct. H. and E. ×95.
FIG. 20.—Fibrosarcoma. Note dermal appendages included in the tumour. H. and VG. ×18.
FIG. 21.—Hair follicles within a fibrosarcoma. H. and VG. ×140.
FIG. 22.—Cystic dilatation of Zymbal's gland associated wlth squamous metaplasia. H. and E. ×32.

1

2

3

4

5

6

Howell.

7

8

9

10

11

12

Howell.

13

14

15

16

17

18

19

20

21

22

Howell.

litters and one had a single litter. Two rats had died early as a result of sepsis in the genital tract. Three animals developed breast tumours ; one of these, found at 11 months, was on the side with intact nipples in a rat that had had 5 litters. The other 2 animals developed breast tumours at 7·75 months on the side from which the nipples had been removed. One of these rats had 3 separate breast tumours and had had 3 litters ; the other rat with a single breast tumour had had 4 litters. Two rats had skin tumours, and one had a squamous carcinoma of the ear duct.

### *Pathology*

### *Normal female and male rat breast*

The adult virgin female rat breast has well developed ducts within the lumen of which is frequently rather dense eosinophilic secretion. The ducts are lined internally by a single layer of cuboidal cells, external to which is a layer of myoepithelium surrounded by a fibrous mantle which decreases in thickness as the ducts branch and become smaller (Fig. 1). Elastic fibres are present within the fibrous tissue encircling the ducts, and by definition a duct must have elastic fibres in its wall (Fig. 2). Arising from the ducts are small bud-like projections forming acini frequently with ill-defined lumina. The acinar cells, and frequently the cells lining the ducts, may contain secretory vacuoles.

The male rat breast compared with other male rodents shows an unusual degree of development throughout adult life. There are fewer ducts as compared with the female, but solid acini are more numerous, the acinar cells being large and eosinophilic (Fig. 3). Both ducts and acini contain eosinophilic secretion, but no evidence of secretory activity was observed in the lining cells.

### *Histology of breasts of DMB-treated animals without tumours*

Ovariectomy made comparatively little difference to the histological structure of the breast except that duct and acinar development was slightly less than in the intact rat. The cells forming the acini were larger and the cytoplasm was more eosinophilic than in the intact rat and they showed no evidence of secretory activity. Castration was also without marked effect on the histological structure of the breast except that in some animals acinar development was less than in the intact male.

Females with hexoestrol implants showed great proliferation and extension of the duct systems, which were also greatly dilated and filled with multiple globules of dense, eosinophilic, sometimes basophilic secretion (Fig. 4). The cells lining many of the ducts showed marked secretory activity. The fibrous stroma of the breast was increased in amount, replacing the normal adipose tissue and the fibrous mantle around the ducts was thicker and rather acellular. Chronic inflammatory cells were present in the stroma. Initially there was also some proliferation of acini, but this was later overshadowed by the duct development. The breast tissues of male animals with hexoestrol implants were similar in appearance to those of the female.

The combination of hexoestrol implants wtih progesterone injections gave the same histological picture in both females and males as with hexoestrol alone, progesterone apparently made no difference to breast histology. The females receiving progesterone injections without hexoestrol showed similar histological appearances to the females without hormonal supplement.

The females that were allowed to breed and lactate during carcinogen treatment showed changes in the breasts dependent upon the stage of pregnancy or lactation at which they were killed or died and no abnormalities of the breast tissues were observed (Fig. 5).

In the rats that had had all the nipples removed from one side of the body, the contralateral breast showed normal pregnancy or lactational changes. On the side with the nipples removed, the breast tissues showed changes of pregnancy or lactation but these were not so well developed as those on the normal side and in some of these animals there was very considerable cystic dilatation of ducts which were filled with globules of dense secretion (Fig. 6).

*Breast tumours*

The breast tumours were first identified as small, discrete, freely mobile nodules in the subcutaneous tissues. They tended to grow fairly rapidly and, if the animal was not killed, they attained a considerable size. They could usually be dissected from the surrounding tissues with ease ; fixation to the deeper tissues was uncommon, but fixation to skin, sometimes associated with ulceration occurred frequently, especially in the larger tumours.

On gross inspection, the tumours had a smooth, sometimes lobulated surface. They were usually yellowish-pink in colour with a soft fleshy consistency, and the reshly cut surface frequently exuded a small quantity of a pale milky fluid. In the larger tumours areas of cystic degeneration with and without haemorrhagic necrosis were common, sometimes with superimposed infective changes.

On histological examination, the breast tumours with a few exceptions were adenocarcinomata, with a complex histological structure which varied not only between tumours but also between different parts of the same tumour.

The tumours showed all degrees of differentiation ; frequently they consisted of solid sheets of cells with scanty stroma and no evidence of differentiation (Fig. 7). Usually however they showed closely applied duct-like structures with a cylindromatous appearance, the intimate structure of which had a solid or cribriform pattern (Fig. 8). Sometimes cysts were present associated with intracystic papillary growth (Fig. 9). Not infrequently areas of solid acinar and trabecular growth were present ; these were usually situated at the periphery of the tumours and the appearances were somewhat similar to the human scirrhous breast carcinoma (Fig. 10). The stroma in these tumours was fairly abundant, consisting of cellular fibrous strands closely investing the epithelial elements. Sometimes, thicker and more hyaline fibrous trabeculae transected the tumour giving rise to a distinctly lobular appearance.

In other types of tumour, alveolar-like growth was observed in which the intimate structure showed a resemblance to the lactating breast, with secretory vacuoles in the epithelial cells lining the alveoli (Fig. 11). The stroma in this type of growth was scanty and consisted of thin fibrous strands separating alveoli from each other, although sometimes coarser bands were also present breaking the tumour into lobules.

Unlike the chemically induced mouse breast tumours squamous metaplasia was rare ; when present it was always adjacent to an area of necrosis or other inflammatory reaction. Metastases in lymph glands or other organs were never observed but invasion of muscle was frequently observed (Fig. 12).

Changes in breast tissue antecedent to tumour formation were rather ill defined. Generally after DMB treatment the fibrous mantle around ducts increased slightly in amount and became thicker. In some instances this was associated with fragmentation and condensation of the elastic fibres around the ducts, so that the elastic tissue appeared as distinct blobs (Fig. 13 and 14). In some ducts there was proliferation of the lining cells, so that these became 2–3 cells thick and almost filled the lumen (Fig. 15 and 16), in certain instances this change progressed to frank intraduct carcinoma sometimes associated with invasion of the duct wall and early infiltration of the stroma (Fig. 17 and 18). Another change consisted of the development of acini which frequently had vacuoles in the lining cells ; these appeared to bud out from ducts and appeared more marked than acinar development in the normal virgin rat breast.

*Other pathological changes*

In addition to breast tumours, certain other neoplastic lesions were found in these rats. The commonest, found in rats of most groups, were skin tumours, which histologically were mainly kerato-acanthomata. They tended to develop later than the breast tumours and were most numerous in animals without breast tumours. In 15 rats, 5 male and 10 female, squamous cell carcinomata arising in the ear ducts were found (Fig. 19). These produced large swellings adjacent to the ears and at post-mortem examination were nearly always secondarily infected. In one of these animals there were massive deposits of secondary squamous cell carcinoma in both lungs, which at first, on gross inspection, was confused with bronchiectasis, but histology showed undoubted carcinoma.

In 12 rats there were lymphomatous lesions, characterized by pallor and enlargement of lymph nodes, thymus, liver and spleen. Histology showed destruction of the normal architecture of these organs with replacement and infiltration by primitive cells, the appearances of which were suggestive of white cell precursors.

In 6 rats, 4 males and 2 females, interesting fibrosarcomatous tumours were found (Fig. 20), consisting of interlacing whorling bundles of collagen which varied considerably in cellularity. Some were hyaline and relatively acellular, but others were extremely cellular, with large, plump fibroblast-like cells showing occasional mitotic figures. Within the tumours there were sometimes occasional epithelial remnants suggestive of breast ducts which appeared to be incorporated by infiltrative and expansive growth of the tumour. Since hair follicles and other skin appendages were sometimes also incorporated (Fig. 21), their presence suggests the tumours arose in tissues more superficial than those of the breast, and from study of early examples of these tumours, they appeared to arise in the dermis immediately subjacent to the epidermis.

Most of the rats, both male and female, receiving hexoestrol implants had enlarged pituitary glands. Histologically these all showed proliferation of basophil cells. No enlargement of the pituitary was observed in rats treated with DMB only.

### DISCUSSION

It is difficult to compare directly the results of these experiments with other reported experiments on the chemical induction of rat breast tumours since the carcinogen and/or its mode of administration differ, as do the various hormone preparations used.

Shay, Harris and Gruenstein (1952) and Shay, Gruenstein and Harris (1956) administered 2 mg. of methylcholanthrene daily by stomach tube to normal female rats and 89 per cent developed breast tumours in an average time of 6·5 months. The age of the animal at the start of treatment had some bearing on tumour yield and induction time, since using older animals, the tumour incidence fell, and the average induction time increased. Employing the same technique but with rats of 42 days old and increasing the daily dose of methylcholanthrene to 10 mg., Huggins, Briziarelli and Sutton (1959) obtained breast tumours in every animal treated in a mean time of 55·9 days. This represents one of the most rapid methods of obtaining the tumours. These results can be compared with those obtained by applying DMB to the skin ; with a 0·5 per cent solution of the carcinogen, 75 per cent developed breast tumours in an average time of 7·25 months, whereas using a 1·6 per cent solution, the average induction time fell to 4·75 months although the tumour incidence was unaltered (Howell, 1959). Geyer *et al.* (1953) using intravenous DMB also showed that the quantity of carcinogen administered influenced the yield and induction time of tumours.

In the present experiments DMB-treated normal male rats did not develop breast tumours. Geyer *et al.* (1951) also failed to obtain breast tumours in males following intravenous DMB, as did Dao and Sunderland (1959), and Kim and Furth (1960) giving intragastric methylcholanthrene. Shay *et al.* (1952) found that 43 per cent of normal males developed breast tumours in 12·8 months, but only one was a typical adenocarcinoma, the remainder being spindle cell or collagenous tumours, and in one instance a fibro-adenoma. In 3 intact males in the present experiments fibrosarcomatous tumours were found, but these are not considered to be breast tumours within the context of this paper.

Ovariectomy followed by DMB completely inhibited breast tumour development despite continuation of treatment for an average period of 8 months. Shay *et al.* (1952) obtained breast tumours in 37 per cent of ovariectomized females, but the average induction time was 12·8 months, rising to 15·5 months in older animals. Huggins, Briziarelli and Sutton (1959) also observed a reduction in tumour incidence following ovariectomy.

No breast tumours were produced in males that were castrated then given DMB. No details have been found in the literature concerning the effect of castration on breast tumour production following administration of carcinogenic hydrocarbons except for Dao and Sunderland (1959) who obtained tumours in 2 castrated animals.

When intact females were treated with hexoestrol implants concurrently with the carcinogen only 2 out of 7 rats developed tumours. Although these observations are based on a small number of animals they tend to support Shay *et al.* (1956) who found that oestrogen caused a sharp fall in tumour incidence and prolongation of average induction time and they made the interesting suggestion that the fall in tumour incidence might be due to development of oestrogen induced pituitary tumours with consequent " physiological hypophysectomy ", thus rendering the animals insensitive to the carcinogen. In this connection it is significant that hypophysectomy completely inhibited breast tumour development following intramuscular injections of DMB (Noble and Walters, 1954) and also following intragastric methylcholanthrene (Huggins, Grand and Brillantes, 1959).

Intact DMB-treated males with hexoestrol implants did not develop breast tumours, but Shay *et al.* (1956) obtained them in 91 per cent of oestrogenized, methylcholanthrene-treated males after 7·5 months ; castration before oestrogen implantation made no significant difference to the tumour incidence.

It should be mentioned that in the present experiments a small group of male and female rats received hexoestrol implants in exactly the same way as those given the carcinogen, but no breast tumours were observed over a period of 18 months.

Progesterone injections plus DMB were given to female rats only, and 3 out of 6 animals developed tumours in 7·8 months, however the dose of progesterone used was substantially less than in other experiments reported in the literature. Shay *et al.* (1952) with a 50 mg. progesterone implant observed a moderate fall in tumour incidence with a slightly prolonged average induction time. However, Huggins, Grand and Brillantes (1959) giving 4 mg. of progesterone daily, obtained breast tumours in all the animals treated with a reduction in average induction time which was even greater using the synthetic preparation 9–2 Bromo-11-ketoprogesterone.

The combination of hexoestrol, progesterone and DMB gave breast tumours in 5 out of 9 female rats after an average induction time of 7 months. Unlike the groups given the hormones separately, the incidence of breast tumours approached that observed in rats treated with DMB alone, although the induction time was 2·25 months longer. Scholler and Carnes (1958) found no effect on tumour production when intravenous DMB was given with oestrogen and progesterone.

Nine male rats were treated for an average period of 7·5 months with the combined hormones and DMB ; one animal, the sole survivor, developed a breast tumour after 9 months. This suggests that by suitable hormonal preparation breast tumours can be induced in the male. In this respect it has been shown that the relative proportion of oestrogen to progesterone is of crucial importance in the development of the normal breast (Folley, 1952) and hence the proportions of the hormones given to animals in carcinogenesis experiments may be of similar significance.

It is commonly accepted that the incidence of carcinoma of the breast in the human female is reduced by breast feeding, and experimentally, Marchant (1958) has shown that breeding with lactation reduces the incidence of chemically induced breast tumours in IF mice. In the present experiments, breeding, lactation, and DMB treatment were continued for an average of 8·8 months but only one of 11 rats developed a tumour, found at 6 months ; this was a fibro-adenoma in contrast to the adenocarcinoma usually induced. Dao and Greiner (1960) using intragastric methylcholanthrene also observed a marked fall in tumour incidence when rats were allowed to breed and suckle their young.

Breast tumour development following skin application of DMB is probably due to a systemic effect caused by absorption of the compound, since it is equally effective in producing breast tumours when given by intranasal instillation (Howell, unpublished observations). One of the factors inhibiting breast tumour development in lactating animals may be that the carcinogen is excreted in the milk leaving insufficient time for it to act on the breast tissues. Shay *et al.* (1950) have shown that methylcholanthrene is excreted in the milk, and in the present experiments milk expressed from lactating breasts of DMB-treated animals fluoresced in ultraviolet light.

In Group 7 the nipples were removed from one side of the body to determine whether inhibition of tumour development in lactating rats was due to the hormonal status and milk excretion, a technique described by Marchant (1959) using IF mice. The results however are inconclusive since only 3 animals developed tumours. Two tumours, found at 7·25 months, were on the side from which the nipples had been excised ; the third rat had a tumour on the normal side but this did not develop until 10 months. It may also be significant that one of the animals with tumours on the side without nipples developed 3 distinct tumours, all found at the same time.

The ear duct tumours observed in 15 animals were all squamous cell carcinomata apparently arising in Zymbal's gland, a sebaceous gland situated near the tympanic membrane. Cystic dilatation of the gland associated with squamous metaplasia of the lining epithelium (Fig. 22) preceded tumour development. Similar tumours have been produced by 2-acetylaminofluorene (Bielschowsky 1944, and Skoryna, Ross and Rudis, 1951) and intranasal instillation of DMB (Howell, unpublished observations). Since the breast is a modified sebaceous gland, it is perhaps not surprising that tumours should also arise in Zymbal's gland, although these tumours have not been reported following intragastric methylcholanthrene.

### SUMMARY

1. Experiments are described which show that painting the skin of female rats with an oily solution of DMB is an effective method of inducing breast tumours. It has been shown that hormonal factors are important for tumour development ; ovariectomy, continued breeding and lactation prevent their development and there is a suggestion, based on a small number of animals, that hexoestrol implants with and without progesterone injections reduce the tumour incidence and prolong the induction time.

2. Experiments have shown that male rats do not develop breast tumours, neither do castrated males. Hexoestrol implants were without effect on breast tumour development, but one out of nine male rats given both hexoestrol and progesterone developed a single breast tumour.

3. Certain other neoplastic changes in rats treated with DMB are briefly described.

4. The results of these experiments are compared with other results on the induction of rat breast tumours reported in the literature.

I am grateful to Professor J. W. Orr for helpful advice and criticism. I am also grateful to Dr. A. D. Hudson for help in the early stages of this investigation. My thanks are due to the Birmingham Branch of the British Empire Cancer Campaign and to the United Birmingham Hospitals Endowment Research Fund for financial support.

### REFERENCES

BIELSCHOWSKY, F.—(1944) *Brit. J. exp. Path.*, **25**, 1.—(1947) *Brit. med. Bull.*, **4**, 382.

DAO, T. L. AND GREINER, M.—(1960) *Proc. Amer. Ass. Cancer Res.*, **3**, 103.

*Idem* AND SUNDERLAND, H.—(1959) *J. nat. Cancer Inst.*, **23**, 567.

FOLLEY, S. J.—(1952) In 'Marshall's Physiology of Reproduction'. Ed. Marshall, F. H. A. London (Longmans), p. 546.

GESCHICKTER, C. F.—(1939) *Science*, **89**, 35.

GEYER, R. P., BLEISCH, V. R., BRYANT, J. E., ROBBINS, A. N., SASLAW, I. M., AND STARE, F. J.—(1951) *Cancer Res.*, **11**, 474.
*Idem*, BRYANT, J. E., BLEISCH, V. R., PEIRCE, E. M., AND STARE, F. J.—(1953) *Ibid.*, **13**, 503.
HOWELL, J. S.—(1959) *Acta Un. int. Cancr.*, **15**, 163.
HUGGINS, C., BRIZIARELLI, G., AND SUTTON, H.—(1959) *J. exp. Med.*, **109**, 25.
*Idem*, GRAND, L. C., AND BRILLANTES, F. P.—(1959) *Proc. nat. Acad. Sci., Wash.*, **45**, 1294.
KIM, U. AND FURTH, J.—(1960) *Proc. Amer. Ass. Cancer Res.*, **3**, 125.
MACKENZIE, I.—(1955) *Brit. J. Cancer*, **9**, 284.
MARCHANT, J.—(1958) *Ibid.*, **12**, 55.—(1959) *Nature, Lond.*, **183**, 629.
MOON, H. D., SIMPSON, M. E., LI, C. H. AND EVANS, H. M.—(1950) *Cancer Res.*, **10**, 549.
NOBLE, R. L. AND WALTERS, J. H.—(1954) *Proc. Amer. Ass. Cancer Res.*, **1**, 35.
SCHOLLER, J. AND CARNES, R. E.—(1958) *Ibid.*, **2**, 343.
SHAY, H., AEGERTER, E. A., GRUENSTEIN, M. AND KOMAROV, S. A.—(1949) *J. nat. Cancer Inst.*, **10**, 255.
*Idem*, FRIEDMANN, B., GRUENSTEIN, M. AND WEINHOUSE, S.—(1950) *Cancer Res.*, **10**, 797.
*Idem*, GRUENSTEIN, M., AND HARRIS, C.—(1956) *Acta Un. int. Cancr.*, **12**, 733.
*Idem*, HARRIS, C., AND GRUENSTEIN, M.—(1952) *J. nat. Cancer Inst.*, **13**, 307.
SKORYNA, S. C., ROSS, R. C., AND RUDIS, L. A.—(1951) *J. exp. Med.*, **94**, 1.
SYMEONIDIS, A.—(1954) *J. nat Cancer Inst.*, **15**, 539.